# RETRACTION: lncRNA OTUD6B‐AS1 Exacerbates As_2_O_3_‐Induced Oxidative Damage in Bladder Cancer via miR‐6734‐5p‐Mediated Functional Inhibition of IDH2

**DOI:** 10.1155/omcl/9894089

**Published:** 2025-12-17

**Authors:** Oxidative Medicine and Cellular Longevity

RETRACTION: Y. Wang, T. Yang, Y. Han, Z. Ren, J. Zou, J. Liu, S. Xi, “lncRNA OTUD6B‐AS1 Exacerbates As_2_O_3_‐Induced Oxidative Damage in Bladder Cancer via miR‐6734‐5p‐Mediated Functional Inhibition of IDH2,” *Oxidative Medicine and Cellular Longevity* 2020, https://doi.org/10.1155/2020/3035624.

The above article, published online on 01 September 2020 in Wiley Online Library (wileyonlinelibrary.com), has been retracted by agreement between the authors; the journal Editor‐in‐Chief, Jeannette Vasquez‐Vivar; and John Wiley & Sons Ltd. The retraction has been agreed due to errors initially raised on PubPeer [1] regarding both Figure 2 and the antibodies used. Specifically:

• The lncRNA panel in Figure 2(i) appears to be a 180 degree rotated duplicate image of the Ki67, MTA1 mimic panel of Figure 8a in [2].
• The specificity of the anti‐Nrf2 was only confirmed through molecular weight, and this caused doubt as to the specificity of the antibody and therefore the rigour of the experiment.

The authors provided a response, however, these did not fully address the underying concerns, and the paper is therefore retracted.

The authors agree with the retraction.
